# Cannabinoid receptor type 1 in the aging gut regulates the mucosal permeability via miR-191-5p

**DOI:** 10.3389/fendo.2023.1241097

**Published:** 2023-08-25

**Authors:** Yunna Lee, Yuju Kim, Soyeong Park, Gwangbeom Heo, Hae Young Chung, Eunok Im

**Affiliations:** ^1^College of Pharmacy, Pusan National University, Busan, Republic of Korea; ^2^Research Institute for Drug Development, Pusan National University, Busan, Republic of Korea

**Keywords:** aging gut, endocannabinoid, CB_1_, miR-191-5p, intestinal permeability

## Abstract

**Background:**

Aging is associated with a broad loss of function throughout the body, and gastrointestinal (GI) dysfunction can occur with aging. The endocannabinoid (eCB) system plays a pivotal role in various GI diseases, and alterations in the eCB system have been observed during brain and skin aging. Therefore, we investigated the putative role of the eCB system in aging-related changes in the intestine.

**Methods:**

The expression of cannabinoid receptor type 1 (CB_1_) was investigated in rat intestinal tissues using quantitative real-time PCR. Cellular senescence was induced by hydrogen peroxide (H_2_O_2_) and hydroxyurea (HU) in rat and human intestinal epithelial cells. Cellular permeability was evaluated by transepithelial electrical resistance (TEER) measurement.

**Results and Discussion:**

The expression of CB_1_ was decreased in the small intestine of aged rats compared to that of young rats. Senescent cells showed reduced TEER values and decreased expression of ZO-1, indicating increased intestinal permeability, which is tightly regulated by the CB_1_ signaling. *In silico* miRNA analysis suggested that ZO-1 was a direct target gene of miR-191-5p. Increased expression of miR-191-5p by HU was restored by CB_1_ agonist ACEA co-treatment. Moreover, NF-κB p65 activation was associated with CB_1_-related miR-191-5p signaling. In conclusion, aging-induced CB_1_ reduction leads to increased intestinal permeability and decreased ZO-1 expression via upregulation of miR-191-5p and NF-κB p65 activation. Taken together, these results suggest that CB_1_ signaling may be a useful strategy to reduce intestinal permeability in aging-related and other inflammatory conditions in the gut.

## Introduction

The increase in the aged population is a global demographic trend. Aging is associated with a physiological decline and widespread loss of function in the body. The gastrointestinal (GI) tract is one of the most complex organ systems, which performs diverse functions that are essential to life. GI disorders due to aging are directly related to the quality of life and morbidity in the older population because multiple functions of the GI tract, such as secretion, digestion, absorption, excretion, and defense, are critical for maintaining general health in the body (1).

One of the important factors that cause age-related intestinal diseases is impaired function of the intestinal mucosal barrier in the elderly population (2). An intact intestinal barrier allows the peaceful coexistence of the intestinal microbiota without an immune response; it induces an effective exchange of molecules between the host and the environment and mediates efficient absorption of nutrients (3). Moreover, the intestinal barrier is the first line of defense against external environments and pathogens, mainly from the food ingestion. In this reason, age-associated modifications of intestinal epithelial barrier leads to the detrimental effects such as a decline of innate immune response to pathogenic stimulus (4). Generally, normal intestinal permeability in healthy individuals can be defined by the proper distribution and location of tight junction proteins, such as zonula occludens-1 (ZO-1), claudins, and occludins (5). However, age-associated changes in tight junction proteins loosen the intestinal barrier, increase intestinal permeability, and elevate the level of pro-inflammatory cytokines (6).

Recent studies have shown that the endocannabinoid (eCB) system plays a key role in regulating intestinal permeability. The eCB system is composed of not only endogenous cannabinoids, such as ligands and two G protein-coupled receptors (GPCRs), including cannabinoid receptor type 1 (CB_1_) and cannabinoid receptor type 2 (CB_2_), but also includes bioactive enzymes associated with the biosynthesis and degradation of eCBs (7). In a study using intestinal epithelial-derived Caco-2 cancer cell lines, the eCB system modulated intestinal permeability by altering the expression levels of ZO-1 and claudin-1 (8). This suggests that eCB system may play a role in modulating intestinal permeability by regulating the expression of tight junction proteins.

Emerging evidence suggests that the eCB system undergoes age-dependent changes. In rodents, mRNA levels of CB_1_ and CB_1_ agonists in the brain are relatively high in adults but markedly reduced in older animals (9). A study on the human brain also indicated that the expression level of the CB_1_ is significantly higher in younger than that in older individuals (10). Age-related reduction in the eCB system has been linked to decreased food intake in aged animals and age-related decline in learning and memory abilities in CB_1_-deficient mouse models (11, 12). As the CB_1_ is also expressed in peripheral organs, it is thought that the eCB system regulates the aging process in extraneural tissues. For example, CB_1_-knockout mice undergo changes that resemble skin aging in their skin structure (13). Therefore, alterations in CB_1_ expression are closely associated with aging in both the brain and peripheral tissues. However, the role of the eCB system in the aging gut has not been studied.

Accordingly, the primary objectives of this study were to (1) investigate the physiological functions of CB_1_ signaling in age-related intestinal alterations, especially focusing on intestinal permeability and (2) to uncover the underlying mechanism by which microRNAs (miRNAs) are involved in CB_1_ signaling in the aged intestine.

## Materials and methods

### Animals

Pathogen-free male Sprague Dawley rats were obtained from Samtako (Osan, South Korea) and fed a standard laboratory diet (Superfeed Co., Wonju, Gangwon, South Korea) *ad libitum*. Animals aged 6 and 24 months were used as young and old rats (n=10), respectively. All animal studies were designed by the Aging Tissue Bank and approved by the Institutional Animal Care Committee of the Pusan National University. We thank the Aging Tissue Bank for providing aging tissues. We followed the guidelines for animal experiments issued by the Pusan National University (Approval Number PNU-2012-0088).

### Cell culture & *in vitro* cell senescence model

Normal rat small intestinal epithelial cells (IEC-6; cat. no. CRL-1592) and the human colonic epithelial adenocarcinoma cell line Caco-2 (cat. no. HTB-37™) were purchased from American Type Culture Collection (Manassas, VA, USA). Cells were cultured in Dulbecco’s modified Eagle’s medium (DMEM) with high glucose (4.5 g/L), 10% heat-inactivated fetal bovine serum, and 100 units/ml penicillin, and 100 μg/ml streptomycin solution (all from Hyclone Laboratories, Logan, UT, USA) at 37 °C in a 5% CO_2_ air environment. The medium was changed every alternate day.

To induce *in vitro* cell senescence, IEC-6 cells were treated with hydrogen peroxide (H_2_O_2_) (Duksan Pure Chemicals, Gyunggi-do, South Korea) for 2 h to induce oxidative stress. H_2_O_2_ was diluted in DMEM to 50, 100, and 150 μM. After treatment with H_2_O_2_ for 2 h, the cultures were rinsed twice with phosphate-buffered saline (PBS) and incubated in DMEM for 24–72 h. To establish another *in vitro* cell senescence model, Caco-2 cells were treated with 150 μM hydroxyurea (HU, cat. no.; H-8627; Sigma-Aldrich, St Louis, MO, USA) for 48 h without changing the culture medium. To inhibit CB_1_ signaling, IEC-6 cells and Caco-2 cells were treated with 2 μM of CB_1_ inverse agonist SR141716A (Tocris Bioscience, Bristol, UK) after H_2_O_2_ exposure in IEC-6 cells and during HU treatment in Caco-2 cells. In contrast, pretreatment with the CB_1_ agonist arachidonyl-2′-chloroethylamide (ACEA) was used to reinforce the agonist activity. Cells were pretreated with 10 μM ACEA (Tocris Bioscience) for 30 min before H_2_O_2_ or HU-induced cell senescence and incubated for 48 h. To measure morphological changes in IEC-6 cells and Caco-2 cells after treatment with H_2_O_2_ and HU, cells were observed under a microscope and photographed using a Nikon ECLIPSE TE 2000-U microscope (Nikon, Tokyo, Japan).

### Senescence-associated β-galactosidase activity

SA-β-gal positive cells were assessed using a senescence detection kit (Cell Signaling Technologies, Danvers, MA, USA), according to the manufacturer’s instructions. Briefly, 1 × 10^5^ cells were seeded in six-well culture plates and stabilized for 48 h. After stabilization, cells were exposed to oxidative stress induced by H_2_O_2_ and HU. After treatment with H_2_O_2_ for 2 h, the cultures were rinsed twice with PBS and incubated in DMEM for 24–72 h. HU treatment was maintained for 6 d to obtain a clearer senescence-like phenotype. After treatment, the cells were washed with PBS and fixed for 15 min in a fixative solution. The fixed cells were then washed twice with PBS and incubated with β-galactosidase staining solution at 37 °C overnight. After staining, images from five random microscopic fields were acquired using a camera-equipped bright-field microscope (Nikon ECLIPSE TE 2000-U microscope). SA-β-gal-positive cells can be easily determined by counting the number of blue cells in the total population.

### Quantitative polymerase chain reaction & reverse transcription PCR

Total RNA was isolated from rat tissues and cells using the TRIzol RNA extraction method (Ambion, Thermo Scientific, Waltham, MA, USA). RNA samples (1,000 ng) were reverse-transcribed to complementary DNA (cDNA) using the RT & GO master mix (MPbio, Santa Ana, CA, USA) and oligo (dT) primers (ELPIS-Biotech, Daejeon, South Korea). To detect CB_1_ and CB_2_ expression in both rat tissues, rat cells and human cells, qPCR was performed using a Thermal Cycler TP800 (TAKARA, Kusatsu, Japan). Primers used for the qPCR were designed manually and the sequences were shown in **Table 1**. After amplification, the results were analyzed using a Thermal cycler dice real time system v5 (TAKARA). The ^ΔΔ^Ct method was used to analyze mRNA expression. RT-PCR was conducted to confirm the expression of tight junction proteins in senescent cells using manually designed primer sequences (**Table 1**). The first cDNA strand was amplified using the Maxime PCR PreMix (iNtRON Biotechnology, Gyeonggi-do, South Korea) with initial denaturation for hot-start (94 °C, 10 min), 35 cycles of denaturation (94 °C, 1 min), primer annealing (53 °C, 1 min), extension (72 °C, 45 s), and final extension (72 °C, 10 min).

**Table 1:** PCR primer sequences.

Genes	Sequences (5’-3’)
**rat CB_1_**	F: GCT GAA CTC CAC CGT GAA CC
	R: CGA AGC ATG TTC CCT TCG TG
**rat CB_2_**	F: CCT TCA CGG CCT CTG TGG
	R: CGG CGT TGA TCT CCT ACC TAC
**rat ZO-1**	F: AGC GAA GCC ACC TGA AGA TA
	R: GAT GGC CAG CAG GAA TAT GT
**rat ZO-2**	F: TAA AGG TGA AAC CGT GAC CA
	R: CAC AGG CCA GGA TGT CTC TA
**rat claudin-2**	F: TCT GGA TGG AGT GTG CGA C
	R: AGT GGC AAG AGG CTG GGC
**rat claudin-4**	F: CTC TCG CCT CCA CGT TAC TC
	R: AGG GTA GGT GGG TGG GTA AG
**rat occludin**	F: CTG TCT ATG CTC GTC ATC G
	R: CAT TCC CGA TCT AAT GAC GC
**rat GAPDH**	F: TGC TGG TGC TGA GTA TGT CG
	R: AGT TGG TGG TGC AGG ATG C
**human CB_1_**	F: TGG AAC TGC GAG AAA CTG CA
	R: ACA GAA GCA GTA CGC TGG TG
**human GAPDH**	F: CGC TCT CTG CTC CTC CTG TT
	R: CCA TGG TGT CTG AGC GAT GT

### Hematoxylin and eosin staining

Segments of the excised colon and small intestine were fixed in 10% buffered formalin solution (Sigma-Aldrich), embedded in paraffin, and stained with H&E. Histological changes were observed using a microscope (Olympus Corp., Tokyo, Japan), photographed with a Moticam 3.0MP Color Digital Camera (Motic, Causeway Bay, Hong Kong), and analyzed using motic images plus 2.0 software.

### Immunohistochemistry and immunofluorescence staining

For IHC staining, slides were baked for 1 h at 60 °C, deparaffinized with xylene (Duksan Pure Chemicals), rehydrated with sequential washes with decreasing concentrations of ethanol (Merck Millipore Corporation, Billerica, MA, USA), and rinsed with tap water (100% xylene 5 min: 2 times, xylene 1:1 with 100% ethanol 5 min: 2 times, 95% ethanol 5 min, 70% ethanol 5 min, 50% ethanol 5 min, tap water). After antigen retrieval and permeabilization, non-specific binding sites were blocked with serum-free blocking buffer (X0909; Agilent, Santa Clara, CA, USA). The slides were then incubated for overnight at 4 °C with 1:50 diluted CB_1_ antibody (Cayman Chemical, Ann Arbor, MI, USA). Antibody binding was detected using a biotinylated secondary antibody and ABC reagent from the Vectastain Elite ABC kit (Vector Laboratories). The slides were developed using peroxidase substrate solution, counterstained with hematoxylin, and mounted using VectaMount mounting medium (all from Vector Laboratories). The slides were then observed and photographed with a Moticam 3.0 MP color digital camera (Motic).

To conduct ZO-1 IF staining, Caco-2 cells were seeded at a density of 1 × 10^4^ cells/well on an eight-well chamber slide (LabTek, Thermo Fisher Scientific). After overnight stabilization, cells were treated with HU (150 μM), SR141716A (2 μM), and ACEA (10 μM) for 24 h. To detect ZO-1 in miRNA mimic- and inhibitor-transfected cells, cells were transfected during the subculture process, seeded on a chamber slide, and incubated for 48 h. After treatment, cells were fixed with 10% neutral buffered formalin solution (Sigma-Aldrich) for 30 min. Fixed cells were washed with PBS three times for 5 min each, and surface tension was reduced using 0.1% Triton X-100 for 1 h at room temperature (RT). Cells were blocked with serum-free blocking buffer (X0909, Agilent) for 1 h, incubated overnight at 4 °C in a humidified chamber with primary antibody, 5 μg/ml of ZO-1 (cat. no. 61-7300; Invitrogen Life Technologies, Gaithersburg, MD, USA). The cells were washed five times for 5 min, each with PBS containing 0.05% Tween 20. After washing, the cells were incubated for 2 h in a 1:1,000 dilution of fluorescein isothiocyanate-conjugated rabbit IgG-heavy and light chain antibody (Bethyl Laboratories, Montgomery, TX, USA). Finally, the cells were washed five times, mounted on slides with VECTASHIELD® mounting medium (Vector Laboratories, Burlingame, CA, USA), and examined with fluorescence microscopy Axioskop FL (Carl Zeiss Meditec, Inc., Dublin, CA, USA) using Metamorph Microscopy Automation and Image Analysis Software (Molecular Devices, Sunnyvale, CA, USA).

### Transepithelial electrical resistance measurement

IEC-6 cells (5 × 10^4^ cells/well) and Caco-2 cells (1 × 10^5^ cells/well) were seeded into the upper chamber of the hanging insert (pore size, 0.4 μm, surface area, 0.33 cm^2^, SPL Life Science, Gyeonggi-do, South Korea) of the 24-well culture plate, while the lower chamber contained the culture medium. Cell culture medium was added to both the apical chamber (200 μl) and basolateral chamber (1 ml). After 5 d, the electrical resistance of the confluent polarized IEC-6 and Caco-2 monolayers was measured by TEER using a Millicell® ERS-2 VoltOhm Meter (Merck Millipore Corporation). Both cells were used in experiments only after the TEER value had increased above 1,000 Ω/cm^2^. IEC-6 cells were treated with H_2_O_2_ for 2 h and washed with fresh culture medium, with or without SR141716A and ACEA. The TEER values were measured at least five time points for 48 h, including 0 h (immediately before H_2_O_2_ treatment) and 2 h (immediately after H_2_O_2_ treatment). Caco-2 cells were treated with HU for 24 h, and at least five time points were measured for the total experimental period. SR141716A treatment was conducted at the same time as HU treatment, whereas ACEA treatment was started 30 min prior to HU treatment. The TEER values for the cell-free control wells were subtracted from the obtained values to remove background values. All TEER measurements were performed in triplicate. The relative TEER value in the transwell plates was calculated as a percentage of the TEER value at 0 h in each group, which was set to 100%.

### Western blotting

Total protein was extracted from tissues and cells using a protein extraction solution (ELPIS Biotech, Daejeon, Republic of Korea). The protein concentration of the lysates was determined using the Pierce BCA Protein Assay Kit (Thermo Fisher Scientific), according to the manufacturer’s instructions. Equal amounts (20 μg) of proteins from each group were then boiled at 90 °C for 5 min in Laemmli sample buffer (ELPIS-Biotech, Daejeon, South Korea) at a volume ratio of 4:1. Total protein was separated by 10% sodium dodecyl sulfate-polyacrylamide gel electrophoresis and transferred to polyvinylidene fluoride membranes (Merck Millipore Corporation) using a wet transfer system (Hoefer Scientific, Holliston, MA, USA). The membranes were blocked with 5% skimmed milk (BD Biosciences, San Jose, CA, USA) in Tris-buffered saline with 0.05% Tween 20 (20 mM Tris, 140 mM sodium chloride, pH 7.5, and 0.05% Tween 20; Amresco, Solon, OH, USA) for 1 h at RT. The membranes were then incubated overnight at 4 °C with the primary antibodies at a 1:1,000 dilution: CB_1_ (cat. no. sc-20754; Santa Cruz Biotechnology Inc., Santa Cruz, CA, USA), and p-p65 NF-κB (cat. no. 3033; Cell Signaling Technology, Inc.), and p-STAT3 (cat. no. 9131; Cell Signaling Technology, Inc.), and STAT3 (cat. no. 9132; Cell Signaling Technology, Inc.) and β-actin (cat. no.; A5441, 1:10,000, Sigma-Aldrich). After incubation, the membranes were washed five times for 10–15 min with Tris-buffered saline containing 0.05% Tween 20, and then incubated with horseradish peroxidase-conjugated anti-rabbit (cat. no. ADI-SAB-300-J), or anti-mouse (cat. no. ADI-SAB-100-J) antibody (1:10000) (both from Enzo Life Sciences, Farmingdale, NY, USA) at RT for 2 h. Antigen–antibody complexes were visualized using enhanced chemiluminescence reagents (WesternBright ECL, Advansta Corporation, San Jose, CA, USA). Densitometric analyses of protein expression were performed using ImageJ 1.47 software.

### miRNA analysis and transfection

Total RNA was isolated using a Hybrid-R™ miRNA isolation kit (GeneAll Biotechnology Co. Ltd, Seoul, South Korea). To quantify miRNA transcript levels, qPCR was performed using the miRCURY LNA miRNA PCR Starter Kit (Qiagen), following the manufacturer’s instructions. The primer sequences used to detect miR-191-5p and the endogenous control miR-103a-3p were as follows: hsa-miR-191-5p (5′- CAA CGG AAU CCC AAA AGC UG -3′) and hsa-miR-103a-3p (5′- AGC AGC AUU GUA CAG GGC UAU GA -3′).

Caco-2 cells were transfected at 70–80% confluence in a six-well plate using Lipofectamine RNAiMax (Invitrogen Life Technologies), following the manufacturer´s instructions. Briefly, 25 pmol/well Lipofectamine RNAiMAX reagent was diluted in Opti-MEM medium (Gibco BRL, Gaithersburg, MD, USA), and 30 pmol/well siRNA, miRNA mimic, and inhibitor were diluted in Opti-MEM medium. Next, the diluted siRNA and diluted Lipofectamine RNAiMAX reagent were mixed in a 1:1 ratio and incubated for 5 min. A mixture of siRNA-lipid complexes was added to the cells. For miR-191a-5p regulation, 50 nmol/L of miR-191-5p mimic and inhibitor (Qiagen) and their negative controls were used (Bioneer, Daejeon, South Korea). STAT3 inhibition was achieved by transfection with 30 pmol/L STAT3 siRNA or a negative control siRNA (Bioneer). Forty-eight hours after transfection, the cells were trypsinized, harvested, and used for experiments.

### Statistical analysis

Statistical analyses were performed using GraphPad Prism version 5.03 for Windows (GraphPad Software, La Jolla, CA, USA). Data are presented as mean ± SD. Student’s *t-test* or one-way or two-way analysis of variance was used to assess statistical significance; p < 0.05 was considered statistically significant.

## Results

### CB_1_ expression was decreased by aging in the small intestine

**Figure 1:**
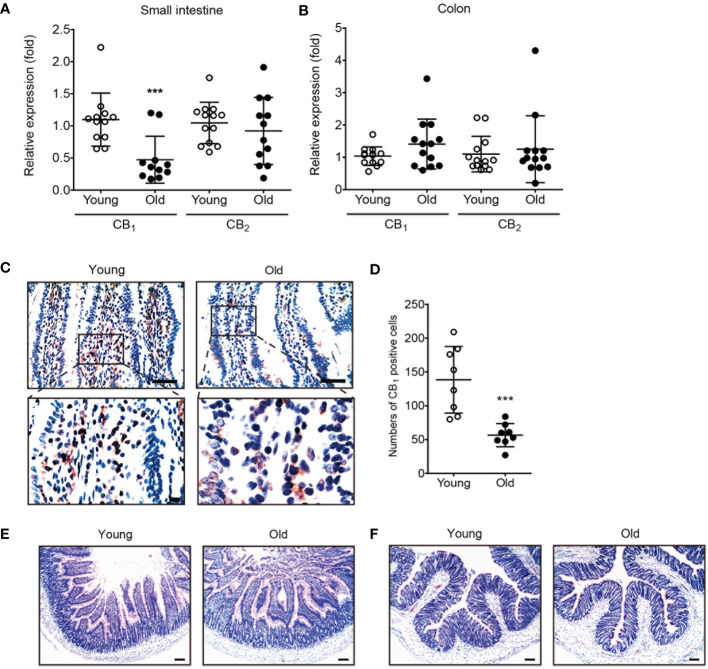
The expression of CB_1_ was decreased in old rat small intestine. **(A, B)** qPCR was conducted to detect RNA expression levels of CB_1_ and CB_2_ in both young (white dot) and old (black dot) tissues of the small intestine **(A)** and colon **(B)**. Statistical differences were determined using *t-test*; triple asterisks (***) indicate p<0.001. Results are presented as means ± SEMs. n=10-12 per group. GAPDH was used for the loading control. **(C)** Immunohistochemistry staining was used to detect CB_1_ expression in the small intestine of young and old rats. Scale bar, 50 μm. **(D)** The graph shows the result of quantitative analyses by using counting of CB_1_ positive cells in each phase. (***) indicate p<0.001. Results are presented as means ± SEMs. n=8 per group. **(E, F)** Small intestine **(E)** and colon **(F)** tissue sections from young and old rats were stained with H&E. No significant change in histology was observed in either the small intestine or colon. Scale bar, 100 μm.

To investigate age-related changes in the expression of eCB receptors, qPCR was conducted using tissues of the small intestine and colon from young and old rats. Interestingly, CB_1_ expression was significantly decreased only in the aged small intestine (**Figure 1A**). In the colon, CB_1_ expression did not decrease significantly (**Figure 1B**). The expression of CB_2_ did not change in either the small intestine or the colon (**Figures 1A, B**). Moreover, IHC staining showed that CB_1_ expression decreased in the small intestine tissues of old rats compared to those of young rats (**Figures 1C, D**). Histological analysis showed no significant changes in the old small intestine and colon compared to the young small intestine and colon (**Figures 1E, F**).

### CB_1_ expression was decreased by H_2_O_2_-induced cellular senescence

**Figure 2:**
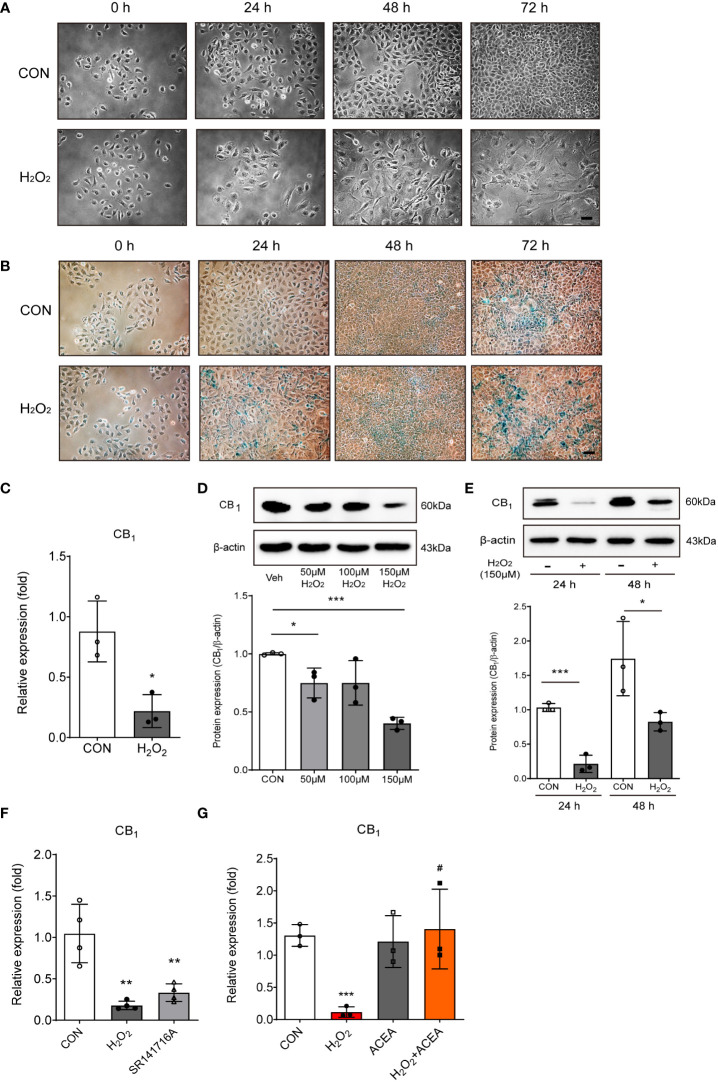
H_2_O_2_-induced cell senescence *in vitro* model induced CB_1_ reduction. **(A)** Morphological changes showed IEC-6 cell senescence by H_2_O_2_ treatment (150 μM) in a time-dependent manner (n=3). Scale bar, 100 μm. **(B)** β-gal staining indicated that positive cells (blue) increased with H_2_O_2_ treatment (150 μM) in a time-dependent manner (n=5). Scale bar, 100 μm. **(C)** qPCR was conducted to confirm the RNA expression of CB_1_ in H_2_O_2_-treated IEC-6 cells. Statistical differences were determined using *t-test;* a single asterisk (*) indicates p<0.05 compared to the control group. Error bars, SD (n=3). GAPDH was used for the loading control. **(D, E)** Western blot analysis was conducted to investigate the CB_1_ expression alteration by H_2_O_2_ treatment. The bar graphs represent the optical density quantification of the protein bands for each group (**D, E,** bottom). Statistical differences were determined using *t-test;* a single asterisk (*) indicates p<0.05, triple asterisks (***) indicate p<0.001 compared to control group. Error bars, SD (n=3). β-actin was used for the loading control. **(F, G)** CB_1_ mRNA expression was detected by qPCR in H_2_O_2_-treated IEC-6 cells and in cells treated with the selective CB_1_ inverse agonist, SR141716A **(F)**, and/or the selective CB_1_ agonist ACEA **(G)**. Statistical differences were determined using *t-test;* **p<0.01 and ***p<0.001 compared to control group. #p<0.05 compared to H_2_O_2_-treated group. Error bars, SD (n=3).

An *in vitro* model of the aged small intestine was produced by treating IEC-6 cells (a rat intestinal epithelial cell line) with H_2_O_2_, because H_2_O_2_ exposure is known to induce cellular senescence. Cell morphology of IEC-6 cells were enlarged, flattened, and irregular in shape following H_2_O_2_ exposure in a time-dependent manner (**Figure 2A**). We also conducted SA-β-gal staining, which is a widely used biomarker for aging cells, to confirm the β-galactosidase activity of senescent cells. H_2_O_2_-treated IEC-6 cells had a greater number of β-gal-positive cells than untreated cells at each time point (**Figure 2B**). Next, we confirmed the expression of CB_1_ in the H_2_O_2_-treated *in vitro* aging model. The expression of CB_1_ mRNA significantly decreased in H_2_O_2_-treated IEC-6 cells (**Figure 2C**). The protein levels of CB_1_ were also decreased by H_2_O_2_ exposure in a concentration- and time-dependent manner (**Figures 2D, E**). Moreover, the selective CB_1_ inverse agonist, SR141716A, downregulated CB_1_ mRNA expression, similar to that in the H_2_O_2_-treated IEC-6 cells (**Figure 2F**). In addition, ACEA co-treatment was directly associated with increased CB_1_ expression (**Figure 2G**). ACEA treatment successfully restored CB_1_ expression after H_2_O_2_ treatment (**Figure 2G**). These results showed that CB_1_ expression was decreased in an *in vitro* H_2_O_2_-induced cell senescence model, which is consistent with the *in vivo* aged rat tissues (**Figure 1**).

### Increased intestinal permeability was associated with the reduction of CB_1_ signaling by regulating ZO-1

**Figure 3:**
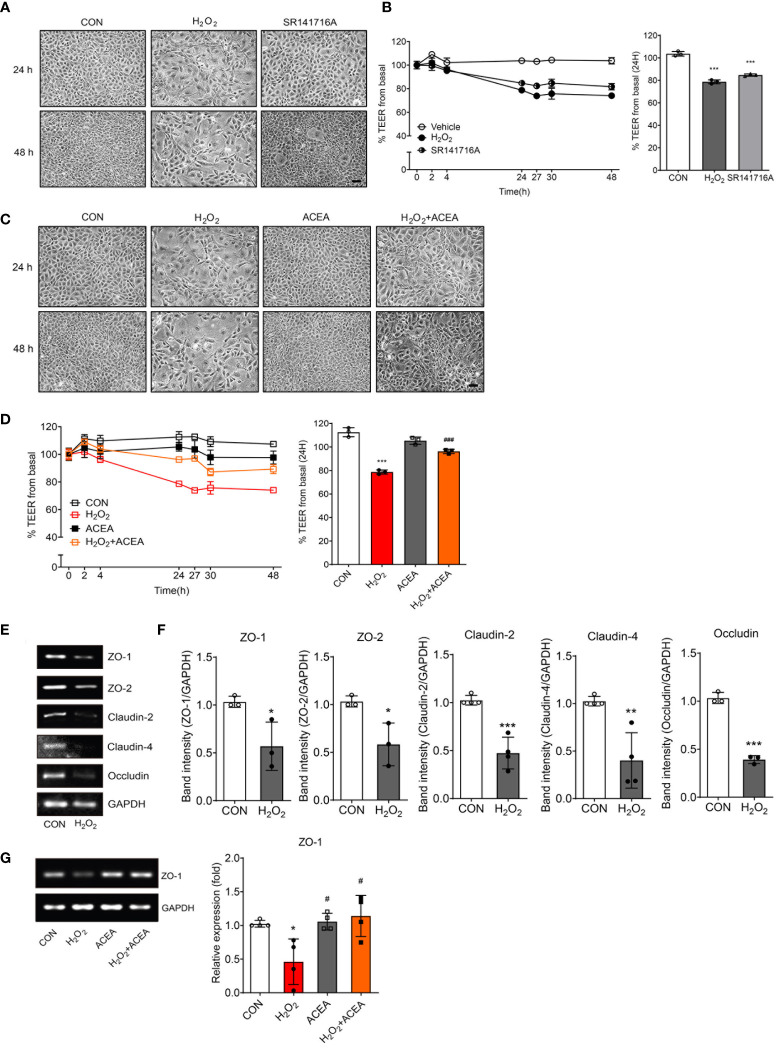
Intestinal permeability and tight junction protein expression were regulated by CB_1_ signaling modulation. **(A)** Morphological changes showed IEC-6 cell senescence by H_2_O_2_ and SR141716A treatment (n=5). Scale bar, 100 μm. **(B)** The TEER values were detected in H_2_O_2_-treated IEC-6 cells and cells treated with the CB_1_ inverse agonist SR141716A. The bar graph indicated the TEER values at 24 h (**B,** right). Statistical differences were determined using *t-test;* ***p<0.001 compared to control group. Error bars, SD (n=3). **(C)** Morphological changes showed IEC-6 cell senescence caused by H_2_O_2_ and the restoration of cell morphology with ACEA treatment after 24 h and 48 h (n=5). Scale bar, 100 μm. **(D)** The decreased TEER values caused by H_2_O_2_ treatment were increased by co-treatment with the CB_1_ agonist ACEA. The bar graph presents the TEER values at 24 h after treatment with H_2_O_2_ and ACEA (**D,** right). Statistical differences were determined using *t-test;* ***p<0.001 compared to control group and ###p<0.001 compared to H_2_O_2_-treated group. Error bars, SD (n=3). **(E, F)** The mRNA expression levels of the tight junction proteins ZO-1, ZO-2, Claudin-2, Claudin-4 and Occludin were detected by RT-PCR in H_2_O_2_-treated IEC-6 cells. RT-PCR results showed a decrease in the levels of these tight junction proteins **(E)** and the bar graph presents the band intensity of the RT-PCR results **(F)**. Statistical differences were determined using *t-test;* *p<0.05, **p<0.01, and ***p<0.001 compared to control group. Error bars, SD (n = 3). GAPDH was used for the loading control. **(G)** Decreased RNA expression of ZO-1 by H_2_O_2_ was restored by ACEA co-treatment. The bar graphs present the band intensity of PCR results. Statistical differences were determined using *t-test;* *p<0.05 compared to control group and #p<0.05 compared to H_2_O_2_-treated group. Error bars, SD (n = 3).

To investigate whether CB_1_ signaling was related to senescent cell phenotypes, we observed cell morphology and measured the TEER value after applying the agonists, SR141716A and ACEA. SR141716A treatment resulted in more aged cell morphology than the untreated control group, although it did not induce morphological changes as much as the H_2_O_2_ treatment (**Figure 3A**). The reduction in TEER value by SR141716A was as strong as that by H_2_O_2_ treatment, and we confirmed that both SR141716A and H_2_O_2_ induced approximately a 20% reduction at 24 h (**Figure 3B**). These results showed that the inhibition of CB_1_ signaling causes intestinal epithelial cells to be considerably similar to the senescent cell phenotype induced by H_2_O_2_ treatment. We used ACEA to confirm whether CB_1_ signaling activation can restore aged cell phenotypes and abnormally increased cell permeability. First, ACEA treatment significantly reduced the morphological alterations in H_2_O_2_-treated senescent cells (**Figure 3C**). The TEER value was significantly reduced by H_2_O_2_ treatment but was considerably restored by ACEA co-treatment (**Figure 3D**). Next, we assessed the alteration of tight junction protein levels in H_2_O_2_-treated IEC-6 cells. H_2_O_2_ treatment considerably downregulated ZO-1, ZO-2, claudin-2, claudin-4, and occludin mRNA expression (**Figures 3E, F**). Moreover, the cells were co-treated with ACEA, and the induction of tight junction protein expression was confirmed. Interestingly, ACEA restored the expression of ZO-1 (**Figure 3G**), but not that of other tight junction proteins. These results suggested that CB_1_ signaling activation restores H_2_O_2_-induced aged cell morphology and reduces intestinal permeability by increasing the expression of tight junction proteins, especially ZO-1.

### Increased intestinal permeability in HU-induced cell senescence was regulated by CB_1_ signaling

**Figure 4:**
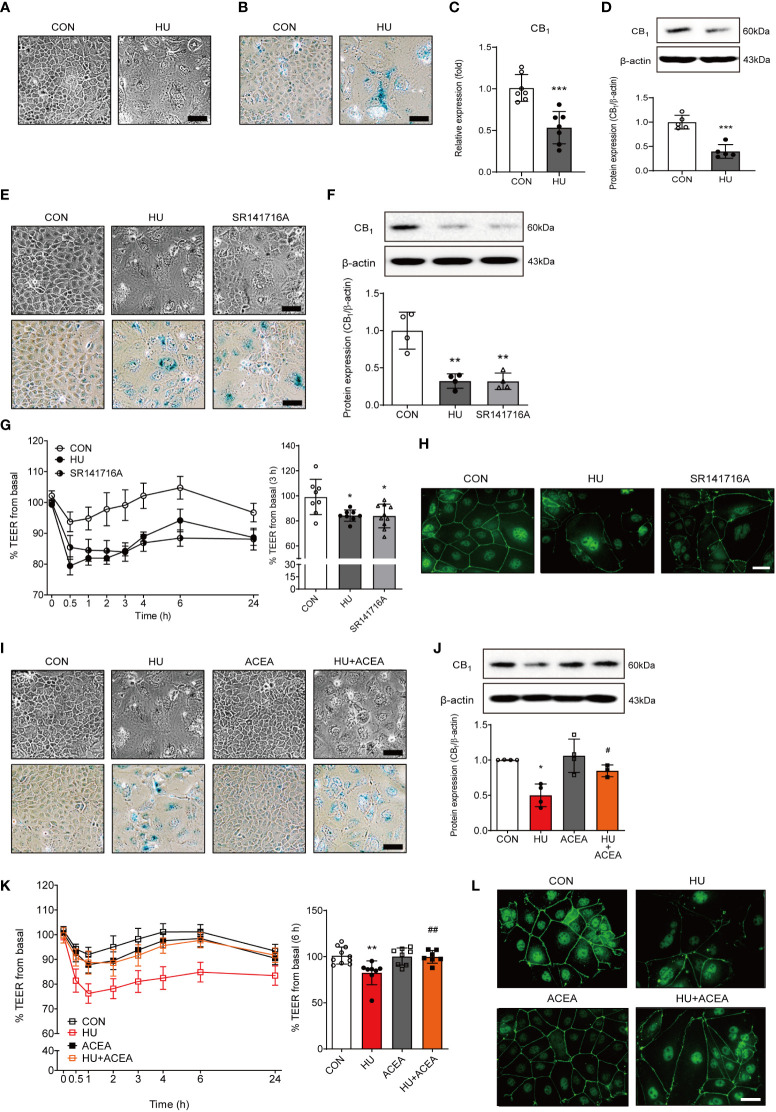
The characteristics of cell senescence and alteration of the TEER value and ZO-1 expression are regulated by CB_1_ signaling. **(A, B)** HU treatment induced morphological changes **(A)** and positive cell intensity in β-gal staining **(B)** in Caco-2 cells. Scale bar, 50 μm. **(C, D)** The alteration of CB_1_ expression was detected by qPCR **(C)** and western blot **(D)**. The bar graph presents the optical density quantification of the protein bands for each group (**D,** bottom). Statistical differences were determined using *t-test;* ***p<0.001 compared to control group. Error bars, SD (n = 3). **(E)** Cell morphology (**E,** upper) and β-gal staining (**E,** bottom) were determined in HU-induced aged cells and SR141716A-treated Caco-2 cells. Scale bar, 50 μm. **(F)** Representative bands from western blot and quantitative analyses of CB_1_ protein expression in Caco-2 cells after HU and SR141716A treatment are shown. β-actin was used as a loading control. Error bars, SD (n = 3). **p<0.01 vs. the control group. **(G)** The effects of HU and SR141716A on Caco-2 cell permeability. Continual resistance values of the Caco-2 cell monolayers are shown. The bar graph presents the TEER values at 3 h after treatment with HU and SR141716A (**G,** right). Statistical differences were determined using *t-test;* *p<0.05 compared to control group. Error bars, SD (n = 8). **(H)** Representative pictures of IF staining of ZO-1 in Caco-2 cells treated with HU and SR141716A. Scale bar, 50 μm. **(I)** Cell morphology (**I,** upper) and β-gal staining (**I,** bottom) in HU-induced aged cells and/or ACEA-treated Caco-2 cells. Scale bar, 50 μm. **(J)** Representative bands of western blot and quantitative analyses of CB_1_ protein expression in Caco-2 cells after HU and/or ACEA treatment are shown. β-actin was used as a loading control. Error bars, SD (n = 3). *p<0.05 compared to control group, #p<0.05 compared to HU treatment group. **(K)** The effects of HU and/or ACEA on Caco-2 cell permeability. Continual resistance values of the Caco-2 cell monolayers are shown. The bar graph presents the TEER values at 6 h after treatment with HU and/or ACEA (**K,** right). Statistical differences were determined using *t-test;* **p<0.01 compared to control group, ##p<0.01 compared to HU treatment group. Error bars, SD (n = 8). **(L)** Representative pictures of IF staining of ZO-1 in Caco-2 cells treated with HU and/or ACEA. Scale bar, 50 μm.

Several recent studies have shown that HU can promote a senescence-like phenotype in various cell types (14–16). To establish new *in vitro* senescence models in intestinal cells, Caco-2 human colonic epithelial adenocarcinoma cells were treated with HU. HU treatment induced senescent cell morphology (**Figure 4A**) and increased β-gal staining intensity (**Figure 4B**). Both the mRNA and protein expression levels of the CB_1_ were significantly decreased in HU-induced Caco-2 cells (**Figures 4C, D**). To confirm whether the regulation of CB_1_ signaling in HU-induced aged cells is similar to that in H_2_O_2_-induced aged cells, SR141716A and ACEA were used to treat HU-induced aged Caco-2 cells. Inhibition of CB_1_ signaling by SR141716A resulted in considerably aged cell phenotypes (**Figure 4E**, top), and increased β-gal-positive cells similar to HU-induced aged cells (**Figure 4E**, bottom). SR141716A treatment significantly decreased CB_1_ expression compared to the untreated control, and the decreased level of expression was similar to HU treatment of Caco-2 cells (**Figure 4F**). The TEER value was significantly lower in both HU-and SR141716A-treated Caco-2 cells than that in non-treated control cells (**Figure 4G**). Moreover, the intensity of IF staining for ZO-1 in tight junctions was clearly diminished in both HU- and SR141716A-treated Caco-2 cells compared to that for ZO-1 in non-treated control cells (**Figure 4H**). Meanwhile, the activation of CB_1_ signaling by co-treatment with ACEA for 6 d after pretreatment for 30 min showed protective effects against HU-induced cell senescence. The ACEA co-treatment group with HU-induced aging cells had a less flattened morphology and slightly decreased β-gal-positive cells compared to HU-induced aging cells (**Figure 4I**). The decreased CB_1_ protein expression after HU treatment was significantly restored by ACEA co-treatment (**Figure 4J**). The TEER value was also increased and normalized by ACEA co-treatment compared to the HU-treated group (**Figure 4K**). Continuous linear staining of ZO-1 was considerably decreased by HU treatment; however, ACEA co-treatment remarkably restored the intensity of ZO-1 staining (**Figure 4L**). These results suggested that the increase in intestinal permeability caused by HU-induced cell senescence is tightly regulated by CB_1_ signaling.

### miR-191-5p regulated intestinal permeability by targeting ZO-1

**Figure 5:**
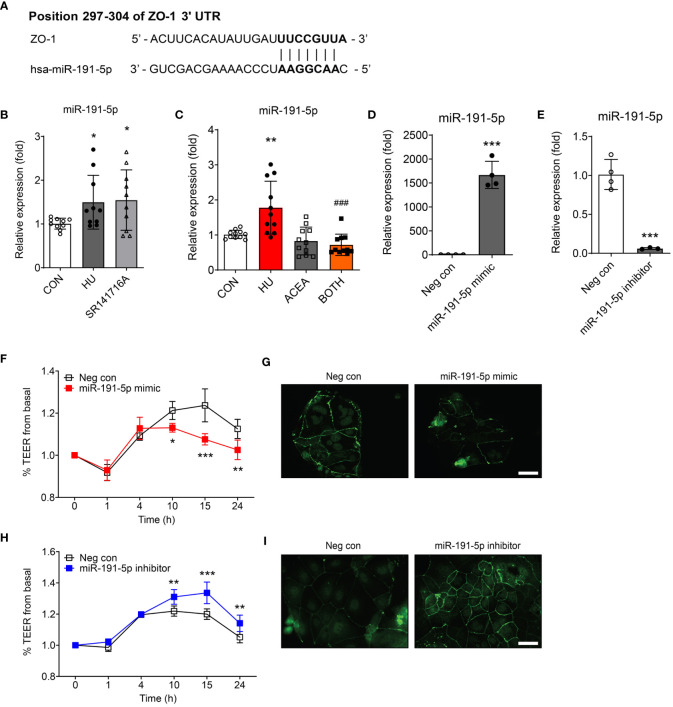
Intestinal permeability was regulated by miR-191-5p targeting ZO-1. **(A)** The likelihood of miRNA targeting ZO-1 was predicted using two independent miRNA target databases. It showed 7–8 mer sequences (black bold) of miR-191-5p binding positions within 3’ UTR of ZO-1 mRNA. **(B, C)** qPCR was used to compare the alteration of miR-191-5p expression after SR141716A **(B)** and ACEA treatments **(C)** to that in the HU-treated aged Caco-2 cells. miR-103a-3p was used as a loading control. Error bars, SD (n = 3). *p<0.05, **p<0.01 vs. the control group, ###p<0.001 vs. the HU treatment group. **(D, E)** The transfection efficacy of miR-191-5p mimic **(D)** and inhibitor **(E)** were tested by qPCR for miR-191-5p expression. Error bars, SD (n = 3). ***p<0.001 vs. the negative control group. **(F-I)** The TEER values were measured to investigate the change of intestinal permeability in Caco-2 cell monolayer by transfection of miR-191-5p mimic **(F)** and inhibitor **(H)**. Statistical differences were determined using *two-way ANOVA;* *p<0.05, **p<0.01, ***p<0.001 vs. the negative control group. Error bars, SD (n = 4). IF staining of ZO-1 was also conducted, and representative pictures were selected of Caco-2 cells transfected with miR-191-5p mimic **(G)** and inhibitor **(I)**. Scale bar, 50 μm.

To identify putative miRNAs, we employed two different computational programs: miRDB (http://mirdb.org) and TargetScan Human Release 8.0 (https://www.targetscan.org), to predict miRNAs containing targeting sites for ZO-1 mRNA. miR-191-5p showed a remarkably high prediction scores in both algorithms, with 88 and 97 points in the miRDB and TargetScan, respectively. These *in silico* analyses revealed that miR-191-5p directly targeted ZO-1 mRNA to inhibit its translation. The 297-304 position in the 3’ UTR of ZO-1 mRNA contained a putative binding site, which is a completely complementary sequence of the seed region of miR-191-5p (**Figure 5A**). Both HU-induced cell senescence and inhibition of CB_1_ signaling by SR141716A significantly increased the expression of miR-191-5p (**Figure 5B**). ACEA co-treatment considerably decreased the expression of HU-induced miR-191-5p (**Figure 5C**). Next, to investigate the functions of miR-191-5p in intestinal permeability, we used an miR-191-5p mimic and inhibitor. Transfection of the miR-191-5p mimic and inhibitor successfully overexpressed and inhibited miR-191-5p expression, respectively (**Figures 5D, E**). Overexpression of miR-191-5p by mimic transfection decreased TEER value and ZO-1 expression (**Figures 5F, G**). In contrast, transfection with the miR-191-5p inhibitor increased the TEER value and ZO-1 expression (**Figures 5H, I**). These results indicated that miR-191-5p is involved in the regulation of intestinal permeability in HU-induced senescent cells by targeting ZO-1.

### Phosphorylation of NF-κB p65 is tightly regulated by CB_1_/miR-191-5p-related signaling in HU-induced aged cells

**Figure 6:**
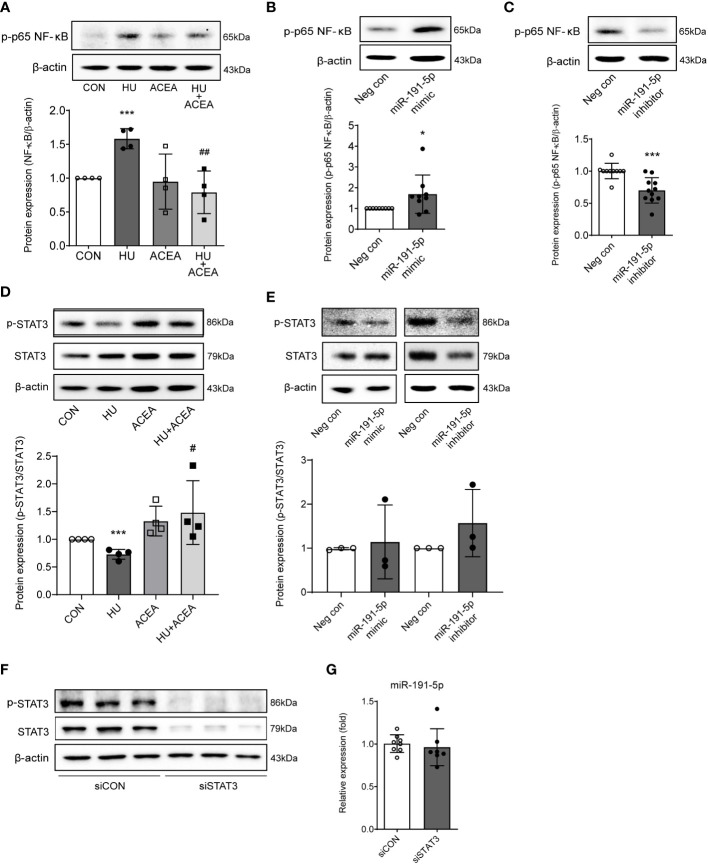
Phosphorylation of NF-κB p65 was modulated by miR-191-5p in HU-induced cell senescence. **(A, D)** Representative bands of western blot and quantitative analyses of phosphorylation of NF-κB p65 **(A)** and p-STAT3 **(D)** protein expression levels in Caco-2 cells after HU and/or ACEA treatment are shown. Error bars, SD (n = 3). ***p<0.001 compared to control group, #p<0.05, ##p<0.01 compared to HU treatment group. **(B, C)** The protein expression level of NF-κB p65 phosphorylation was detected by western blot in miR-191-5p mimic- **(B)** and inhibitor- **(C)** transfected Caco-2 cells. Error bars, SD (n = 3). *p<0.05, ***p<0.001 compared to negative control group. **(E)** Phosphorylation of STAT3 was evaluated with miR-191-5p mimic- and inhibitor-transfected Caco-2 cells. The bar graph below shows the result of quantitative analysis. **(F)** p-STAT and STAT3 protein expression was shown in cells transiently transfected with siRNA of STAT3. **(G)** miR-191-5p expression in transiently transfected Caco-2 cells with siSTAT3 was measured using qPCR. Error bars, SD (n = 3).

Previous reports have showed that the transcriptional regulation of ZO-1-mediated barrier permeability is related to NF-κB signaling and STAT3 signaling (17–19). Also, miR-191 is closely linked to the activation of pro-inflammatory pathways, such as NF-κB signaling (18). To determine the specific miR-191-5p-related molecular regulator upstream of ZO-1, we tested the changes in NF-κB and STAT3 signaling. The phosphorylation of NF-κB p65 was increased and phosphorylation of STAT3 was decreased in HU-induced aged Caco-2 cells, but activation of the CB_1_ by ACEA co-treatment successfully restored these alterations of phosphorylation (**Figures 6A, D**). Transfection of Caco-2 cells with the miR-191-5p mimic increased the phosphorylation of NF-κB p65 (**Figure 6B**), whereas miR-191-5p inhibitor transfection inhibited the phosphorylation of NF-κB p65 (**Figure 6C**). However, phosphorylation of STAT3 did not show any significant changes in either miR-191-5p mimic- or inhibitor-transfected Caco-2 cells (**Figure 6E**). Moreover, the expression of miR-191-5p did not change upon STAT3 knockdown by siSTAT3-transfection in Caco-2 cells (**Figures 6F, G**). These results implied that NF-κB p65 activation is directly regulated by miR-191-5p, but STAT3 signaling is independent of miR-191-5p, even though it is involved in the CB_1_ signaling pathway during cell senescence.

## Discussion

**Figure 7:**
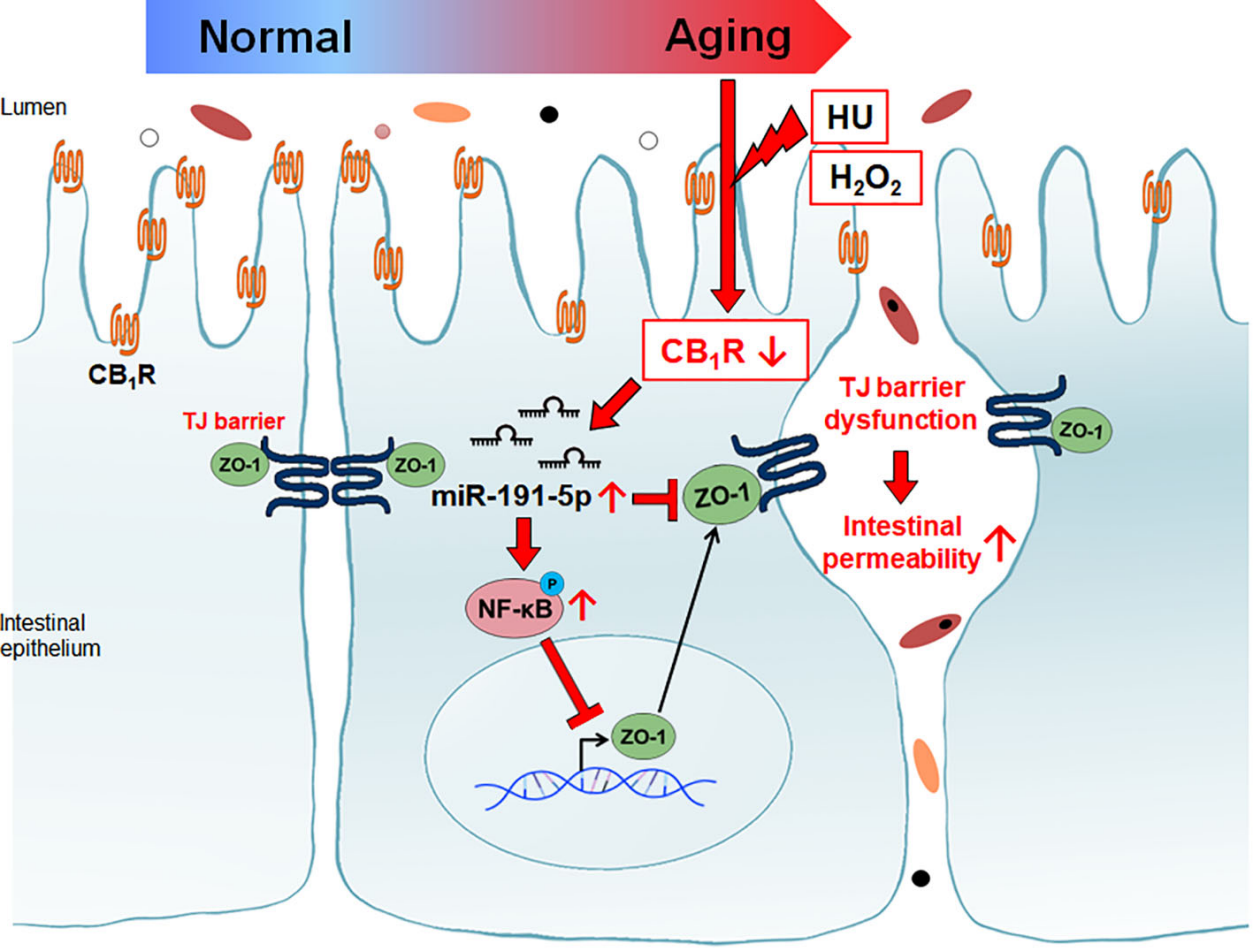
Reduction of CB_1_ in aged intestinal epithelial cells caused tight junction dysfunction. Decreased CB_1_ is associated with the induction of miR-191-5p. miR-191-5p reduced ZO-1 expression by targeting directly and via activation of p-p65 NF-κB transcription factor.

Increased intestinal tight junction permeability in the aging gut is a well-known physiological change, but the reason for this is not yet fully understood. This is the first report indicating that CB_1_ signaling is directly related to aging gut permeability through the downregulation of ZO-1 protein. Our results also demonstrated the detailed mechanisms by which miR-191-5p and NF-κB p65 activation are closely associated with intestinal permeability by the downregulation of ZO-1 in CB_1_ defected aging gut (**Figure 7**).

Our results showed that the expression of the CB_1_ was significantly decreased in the small intestine of old rats compared to that of young rats (**Figure 1A**). To create an *in vitro* model of the aged intestinal epithelium, the expression of CB_1_ was also significantly decreased by H_2_O_2_ and HU treatment compared to the vehicle-treated control in both concentration- and time-dependent manners (**Figures 2**, **4**). Our results are consistent with those of a previous study that showed a reduction in CB_1_ expression in aged human brain tissues (10). The specific reasons and mechanisms underlying the decline in CB_1_ expression during aging remain unknown. One possibility is that the decline in CB_1_ expression is accompanied with alteration in the levels of cannabinoid ligands because it is synthesized and released “on demand” (20). Therefore, decreased levels of endogenous cannabinoid ligands, as well as increased levels of its catabolizing enzymes, can cause a decrease in signaling of the cannabinoid system during aging (20, 21). Previous reports have indicated that CB_1_ exert protective effects against increased intestinal motility and epithelial damage during intestinal inflammation (7). As aging progresses, the expression of the CB_1_ gradually declines, diminishing its protective effect in the gut.

In contrast, the expression level of the CB_2_ showed no significant change with aging (**Figures 1A, B**). The CB_2_ is generally expressed in immune cells, such as macrophages and plasma cells, and is highly associated with inflammation (22). Additionally, the CB_2_ expression is upregulated in the colonic epithelium of patients with inflammatory bowel disease, and it may exert anti-inflammatory effects (23). Therefore, CB_2_ expression may contribute to the regulation of abnormal inflammatory pathogenesis, rather than age-related intestinal physiology. Although further studies are required to investigate more specific functions and characterizations of the eCB system in the aging gut, this is meaningful as the first report to show that alterations in CB_1_ and CB_2_ expression levels in the intestine reflect age-associated changes.

In elderly people, abnormal intestinal permeability, also known as a leaky gut, may be a causative factor driving chronic and difficult-to-treat health problems, and elderly people have been shown to be at greater risk for the development of autoimmune disorders than younger adults (2). Endogenous cannabinoids have the ability to increase permeability, resulting in inflammation, whereas exogenous phytocannabinoids can inhibit or recover cytokine-induced increased permeability (24). Plant-derived synthetic cannabinoids also reduce abnormal intestinal permeability, suggesting their therapeutic potential (8). In murine colitis, cannabinoids decrease histologic and microscopic inflammation (25). Although many studies have discussed the regulation of intestinal permeability through the activation of CB receptors by exogenous or endogenous cannabinoids, there is no evidence regarding the association between age-related intestinal permeability and the eCB system.

In our results, an *in vitro* model of aged intestinal epithelium demonstrated a reversible decrease in TEER (i.e., increased intestinal permeability) over the measurement period. The recovery of morphological changes in cell senescence, as well as TEER values of H_2_O_2_ and HU treatments, was demonstrated by treatment with the CB_1_ selective agonist ACEA (**Figures 3C, D**, **4I, K**). Moreover, treatment with the ACEA upregulated the HU-induced decrease in CB_1_ expression (**Figure 4J**). Our data suggested that increased intestinal permeability in the aging gut is highly associated with decreased expression levels of the CB_1_ and inhibition of CB_1_ signaling. Moreover, reactivation of CB_1_ signaling successfully restored intestinal permeability. These results provide new information regarding the close relationship between CB_1_ signaling and abnormally increased intestinal permeability in the aging gut.

Meanwhile, SR141716A treatment decreased CB_1_ expression to the same extent as HU treatment (**Figure 4F**). Originally, SR141716A was well-described as an antagonist of CB_1_, but it exerts inverse agonist activity by conformational stabilization of the inactive state of CB_1_ (26). The inverse agonist activity of SR141716A directly inhibits the activation of peripheral CB_1_ (27). In our study, SR141716A directly reduced CB_1_ expression without other stimulation. Additionally, the inverse agonism of SR141716A reduced CB_1_ activity, consequently showing similar phenotypes to senescence-induced cells. SR141716A treatment mimicked cell senescence and increased intestinal permeability (**Figures 3A, B**, **4E, G**).

In the regulation of gut barrier function, the eCB system plays an important role as a control tower for gut permeability by altering the distribution of tight junction proteins (28, 29). In our screening data, H_2_O_2_-treated aged IEC-6 showed significantly decreased levels of most tight junction proteins, including ZO-1, ZO-2, claudin-2, claudin-4, and occludin, compared to non-treated vehicle controls (**Figures 3E, F**). Furthermore, activation of CB_1_ signaling by co-treatment with ACEA restored the expression of ZO-1 but not that of other tight junction proteins (**Figure 3G**). In Caco-2 cells, the intensity of IF staining for ZO-1 markedly decreased HU- and SR141716A-treated cells, and co-treatment with ACEA resulted in a slight increase in the expression of ZO-1 (**Figures 4H, L**). These results suggested that ZO-1 plays an important role in regulating CB_1_-related intestinal permeability in the aging gut.

As the last step, we investigated the underlying molecular mechanisms of ZO-1 tight junction proteins in aged intestinal epithelium using miRNA analysis. Some studies have demonstrated that miRNAs are extensively involved in the ZO-1-associated intestinal epithelial barrier function. For example, miR-155 is overexpressed in the intestinal epithelia of severe acute pancreatitis, consequently inhibiting the synthesis of ZO-1 and E-cadherin (30). Overexpression of miR-21 is associated with an impaired intestinal epithelial barrier and decreased ZO-1 and occludin expression levels (31). However, these miRNAs indirectly downregulate ZO-1 by targeting the Rho family of GTPases, such as RhoA and RhoB (30, 31). For this reason, we aimed to find the optimal miRNA satisfying two requirements: 1) targeting ZO-1 directly; 2) associating CB1 signaling. Finally, we selected miR-191-5p as a novel CB_1_ signaling-associated miRNA targeting ZO-1. According to a previous study, miR-191-5p accelerates TNF-α-induced intestinal epithelial injury by ZO-1 depletion in IEC-6 cells (32). Similarly, miR-191-5p activation inhibited ZO-1 expression and decreased the TEER value, suggesting an increase in intestinal permeability in Caco-2 cells (**Figures 5F, G**). Inhibition of miR-191-5p showed the opposite results by increasing ZO-1 expression and TEER value (**Figure 5H, I**). Moreover, the expression of miR-191-5p was increased in HU- and SR141716A-treated senescent Caco-2 cells and decreased in ACEA-co-treated HU-induced aged Caco-2 cells (**Figures 5B, C**). These results indicated that miR-191-5p is highly associated with CB_1_ signaling in senescence-induced cells, suggesting that the regulation of the CB_1_ in aging-induced intestinal permeability is involved in miR-191-5p-dependent signaling.

In conclusion, our data indicated that the reduction of CB_1_ in H_2_O_2_- and HU-treated aging cells causes the induction of miR-191-5p expression, increased NF-κB p65 phosphorylation, decreased ZO-1 expression, and functional opening of the intestinal tight junction tight junction barrier. Therefore, our results implied that modulation of the eCB system is important for the regulation of aging-associated intestinal permeability and inflammation. Understanding the aging gut physiology is important for maintaining healthy conditions in the aged population. This study provides clues for the development of therapeutic adaptation by activating the CB_1_ to treat intestinal disorders in the elderly population. Returning and restoring eCB system homeostasis in the aging gut is essential for improving the quality of life in elderly population.

## Data availability statement

The original contributions presented in the study are included in the article/supplementary materials, further inquiries can be directed to the corresponding author.

## Ethics statement

The animal study was approved by Institutional Animal Care Committee of the Pusan National University (Approval Number PNU-2012-0088). The study was conducted in accordance with the local legislation and institutional requirements.

## Author contributions

YL performed the experiments, analyzed the data and wrote the manuscript. YK, SP, and GH performed the experiments. HC contributed the rat samples and interpreted the data. EI conceived the idea, designed the research, interpreted the data, and wrote the manuscript. All authors contributed to the article and approved the submitted version.

## References

[1] Saffrey MJ. Aging of the mammalian gastrointestinal tract: a complex organ system. Age (Dordr) (2014) 36:9603. doi: 10.1007/s11357-013-9603-2 24352567 PMC4082571

[2] Karper WB. Intestinal permeability, moderate exercise, and older adult health. Holistic Nurs practice. (2011) 25:45–8. doi: 10.1097/HNP.0b013e3181fe26ff 21150504

[3] Maynard CL Elson CO Hatton RD Weaver CT. Reciprocal interactions of the intestinal microbiota and immune system. Nature (2012) 489:231–41. doi: 10.1038/nature11551 PMC4492337 22972296

[4] Nicoletti C. Age-associated changes of the intestinal epithelial barrier: local and systemic implications. Expert Rev Gastroenterol Hepatol (2015) 9:1467–9. doi: 10.1586/17474124.2015.1092872 26395417

[5] Ulluwishewa D Anderson RC McNabb WC Moughan PJ Wells JM Roy NC. Regulation of tight junction permeability by intestinal bacteria and dietary components. J Nutr (2011) 141:769–76. doi: 10.3945/jn.110.135657 21430248

[6] Tran L Greenwood-Van Meerveld B. Age-associated remodeling of the intestinal epithelial barrier. J Gerontol A Biol Sci Med Sci (2013) 68:1045–56. doi: 10.1093/gerona/glt106 PMC3738030 23873964

[7] Lee Y Jo J Chung HY Pothoulakis C Im E. Endocannabinoids in the gastrointestinal tract. Am J Physiol Gastrointestinal liver Physiol (2016) 311:G655–G66. doi: 10.1152/ajpgi.00294.2015 27538961

[8] Alhamoruni A Lee AC Wright KL Larvin M O'Sullivan SE. Pharmacological effects of cannabinoids on the Caco-2 cell culture model of intestinal permeability. J Pharmacol Exp Ther (2010) 335:92–102. doi: 10.1124/jpet.110.168237 20592049

[9] Berrendero F Romero J Garcia-Gil L Suarez I de la Cruz P Ramos JA. Changes in cannabinoid receptor binding and mRNA levels in several brain regions of aged rats. Bba-Mol Basis Dis (1998) 1407:205–14. doi: 10.1016/S0925-4439(98)00042-8 9748581

[10] Glass M Dragunow M Faull RL. Cannabinoid receptors in the human brain: a detailed anatomical and quantitative autoradiographic study in the fetal, neonatal and adult human brain. Neuroscience (1997) 77:299–318. doi: 10.1016/s0306-4522(96)00428-9 9472392

[11] Wang L Liu J Harvey-White J Zimmer A Kunos G. Endocannabinoid signaling via cannabinoid receptor 1 is involved in ethanol preference and its age-dependent decline in mice. Proc Natl Acad Sci United States America. (2003) 100:1393–8. doi: 10.1073/pnas.0336351100 PMC298783 12538878

[12] Bilkei-Gorzo A Racz I Valverde O Otto M Michel K Sastre M. Early age-related cognitive impairment in mice lacking cannabinoid CB1 receptors. Proc Natl Acad Sci United States America. (2005) 102:15670–5. doi: 10.1073/pnas.0504640102 PMC1266095 16221768

[13] Bilkei-Gorzo A Drews E Albayram O Piyanova A Gaffal E Tueting T. Early onset of aging-like changes is restricted to cognitive abilities and skin structure in Cnr1(-)/(-) mice. Neurobiol aging (2012) 33:200.e11–22. doi: 10.1016/j.neurobiolaging.2010.07.009 20724033

[14] Yu Q Cheng X. Hydroxyurea-induced membrane fluidity decreasing as a characterization of neuronal membrane aging in Alzheimer's disease. Aging (Albany NY) (2021) 13:12817–32. doi: 10.18632/aging.202949 PMC8148445 33972461

[15] Kapor S Čokić V Santibanez JF. Mechanisms of hydroxyurea-induced cellular senescence: an oxidative stress connection? Oxid Med Cell Longev (2021) 2021:7753857. doi: 10.1155/2021/7753857 34707779 PMC8545575

[16] Guo Y Tang Z Yan B Yin H Tai S Peng J. PCSK9 (Proprotein convertase subtilisin/kexin type 9) triggers vascular smooth muscle cell senescence and apoptosis: implication of its direct role in degenerative vascular disease. Arterioscler Thromb Vasc Biol (2022) 42:67–86. doi: 10.1161/atvbaha.121.316902 34809446

[17] Fang Y Wu C Wang Q Tang J. Farnesol contributes to intestinal epithelial barrier function by enhancing tight junctions via the JAK/STAT3 signaling pathway in differentiated Caco-2 cells. J Bioenerg Biomembr (2019) 51:403–12. doi: 10.1007/s10863-019-09817-4 31845097

[18] Gu Y Ampofo E Menger MD Laschke MW. miR-191 suppresses angiogenesis by activation of NF-κB signaling. FASEB J Off Publ Fed Am Societies Exp Biol (2017) 31:3321–33. doi: 10.1096/fj.201601263R 28424351

[19] Zhou H Jin C Cui L Xing H Liu J Liao W. HMGB1 contributes to the irradiation-induced endothelial barrier injury through receptor for advanced glycation endproducts (RAGE). J Cell Physiol (2018) 233:6714–21. doi: 10.1002/jcp.26341 PMC13482486 29215715

[20] Tao R Li C Jaffe AE Shin JH Deep-Soboslay A Yamin R. Cannabinoid receptor CNR1 expression and DNA methylation in human prefrontal cortex, hippocampus and caudate in brain development and schizophrenia. Transl Psychiatry (2020) 10:158. doi: 10.1038/s41398-020-0832-8 32433545 PMC7237456

[21] Li R Huang Z Luo J Luo H Wang W. Downregulation of the CB1-mediated endocannabinoid signaling underlies D-galactose-induced memory impairment. Front Mol Neurosci (2020) 13:130. doi: 10.3389/fnmol.2020.00130 32848596 PMC7399637

[22] Wright K Rooney N Feeney M Tate J Robertson D Welham M. Differential expression of cannabinoid receptors in the human colon: cannabinoids promote epithelial wound healing. Gastroenterology (2005) 129:437–53. doi: 10.1016/j.gastro.2005.05.026 16083701

[23] Izzo AA. The cannabinoid CB(2) receptor: a good friend in the gut. Neurogastroenterol Motil. (2007) 19:704–8. doi: 10.1111/j.1365-2982.2007.00977.x 17727390

[24] Alhamoruni A Wright KL Larvin M O'Sullivan SE. Cannabinoids mediate opposing effects on inflammation-induced intestinal permeability. Br J Pharmacol (2012) 165:2598–610. doi: 10.1111/j.1476-5381.2011.01589.x PMC3423254 21745190

[25] Feng YJ Li YY Lin XH Li K Cao MH. Anti-inflammatory effect of cannabinoid agonist WIN55, 212 on mouse experimental colitis is related to inhibition of p38MAPK. World J Gastroenterol WJG (2016) 22:9515–24. doi: 10.3748/wjg.v22.i43.9515 PMC5116595 27920472

[26] Shim JY Bertalovitz AC Kendall DA. Probing the interaction of SR141716A with the CB1 receptor. J Biol Chem (2012) 287:38741–54. doi: 10.1074/jbc.M112.390955 PMC3493917 22995906

[27] Gross A Terraza A Marchant J Bouaboula M Ouahrani-Bettache S Liautard JP. A beneficial aspect of a CB1 cannabinoid receptor antagonist: SR141716A is a potent inhibitor of macrophage infection by the intracellular pathogen Brucella suis. J leukocyte Biol (2000) 67:335–44. doi: 10.1002/jlb.67.3.335 10733093

[28] Everard A Cani PD. Diabetes, obesity and gut microbiota. Best Pract Res Clin gastroenterol (2013) 27:73–83. doi: 10.1016/j.bpg.2013.03.007 23768554

[29] Muccioli GG Naslain D Backhed F Reigstad CS Lambert DM Delzenne NM. The endocannabinoid system links gut microbiota to adipogenesis. Mol Syst Biol (2010) 6:392. doi: 10.1038/msb.2010.46 20664638 PMC2925525

[30] Tian R Wang RL Xie H Jin W Yu KL. Overexpressed miRNA-155 dysregulates intestinal epithelial apical junctional complex in severe acute pancreatitis. World J Gastroenterol WJG (2013) 19:8282–91. doi: 10.3748/wjg.v19.i45.8282 PMC3857451 24363519

[31] Yang Y Ma Y Shi C Chen H Zhang H Chen N. Overexpression of miR-21 in patients with ulcerative colitis impairs intestinal epithelial barrier function through targeting the Rho GTPase RhoB. Biochem Biophys Res Commun (2013) 434:746–52. doi: 10.1016/j.bbrc.2013.03.122 23583411

[32] Wang L Zhang R Chen J Wu Q Kuang Z. Baicalin Protects against TNF-α-Induced Injury by Down-Regulating miR-191a That Targets the Tight Junction Protein ZO-1 in IEC-6 Cells. Biol Pharm Bull (2017) 40:435–43. doi: 10.1248/bpb.b16-00789 28111380

